# Supplementary material for: Glyoxylate Shunt and Pyruvate-to-Acetoin Shift Are Specific Stress Responses Induced by Colistin and Ceragenin CSA-13 in Enterobacter hormaechei ST89
Source: Microbiol Spectr. 2023 Jun 20;11(4):e01215-23. doi: 10.1128/spectrum.01215-23 (PMC10434160; doi:10.1128/spectrum.01215-23)
Supplement: Supplemental file 1 — Fig. S1 and S2. Download spectrum.01215-23-s0001.pdf, PDF file, 5.3 MB [file spectrum.01215-23-s0001.pdf]

Glyoxylate shunt and pyruvate to acetoin shift are specific stress responses induced by colistin and ceragenin CSA-13 in *Enterobacter hormaechei* ST89

Suhanya V. Prasad[1], Krzysztof Fiedoruk[1], Magdalena Zakrzewska[1], Paul B. Savage[2], Robert Bucki[1]

[1] Department of Medical Microbiology and Nanobiomedical Engineering, Medical University of Bialystok, Bialystok, Poland.

[2] Department of Chemistry and Biochemistry, Brigham Young University, Provo, UT 84601, USA.

Supplemental material

Figure S1 and S2

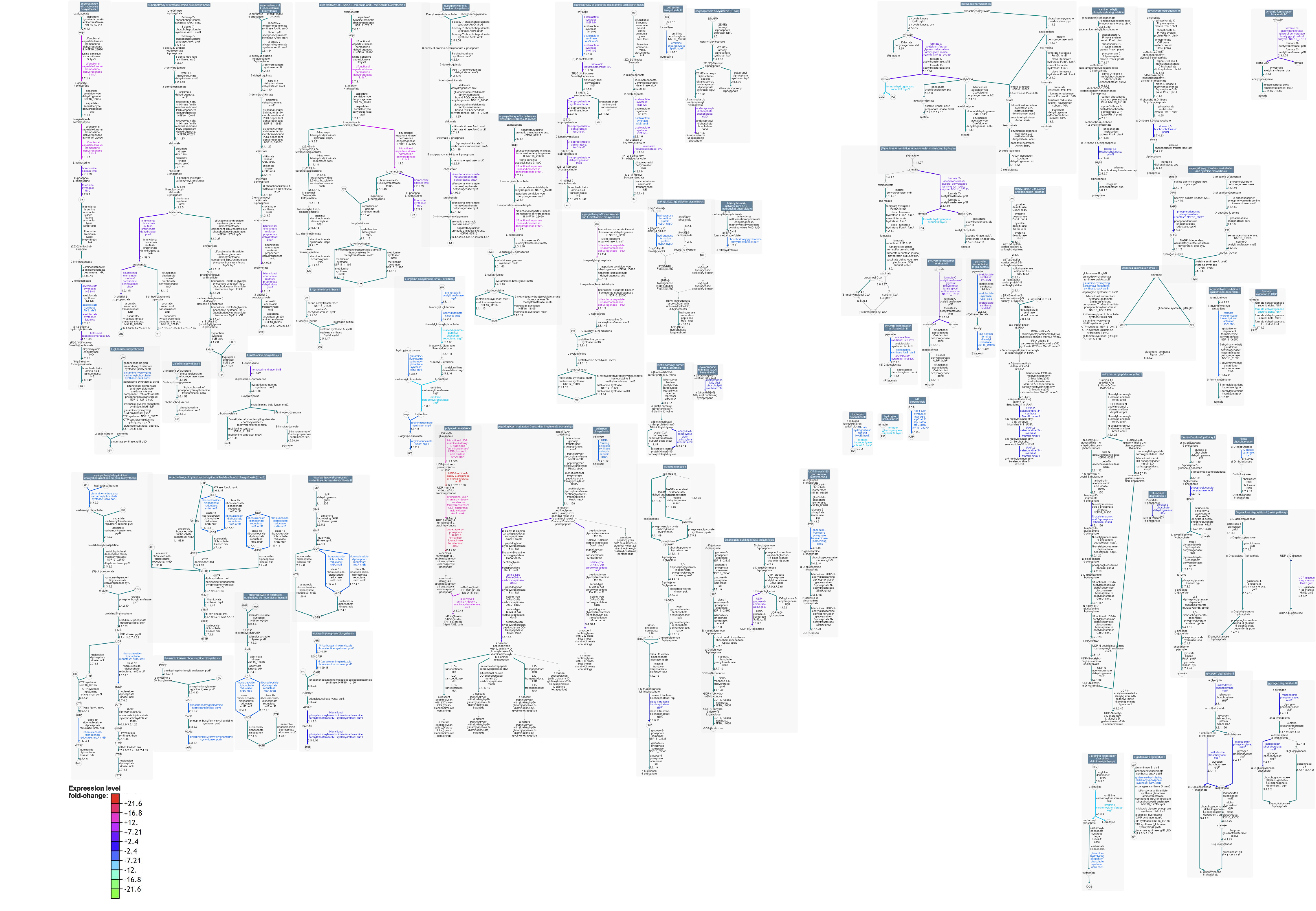

FIG S1 Metabolic pathways affected by the differentially expressed genes (DEGs) genes in Eh4236Csa13R isolate; the level of down- and up-regulation is shown by the heatmap from green, through blue, to red colour, respectively. The analysis and results visualization was performed with the Pathway Tools v26.0 software (16).

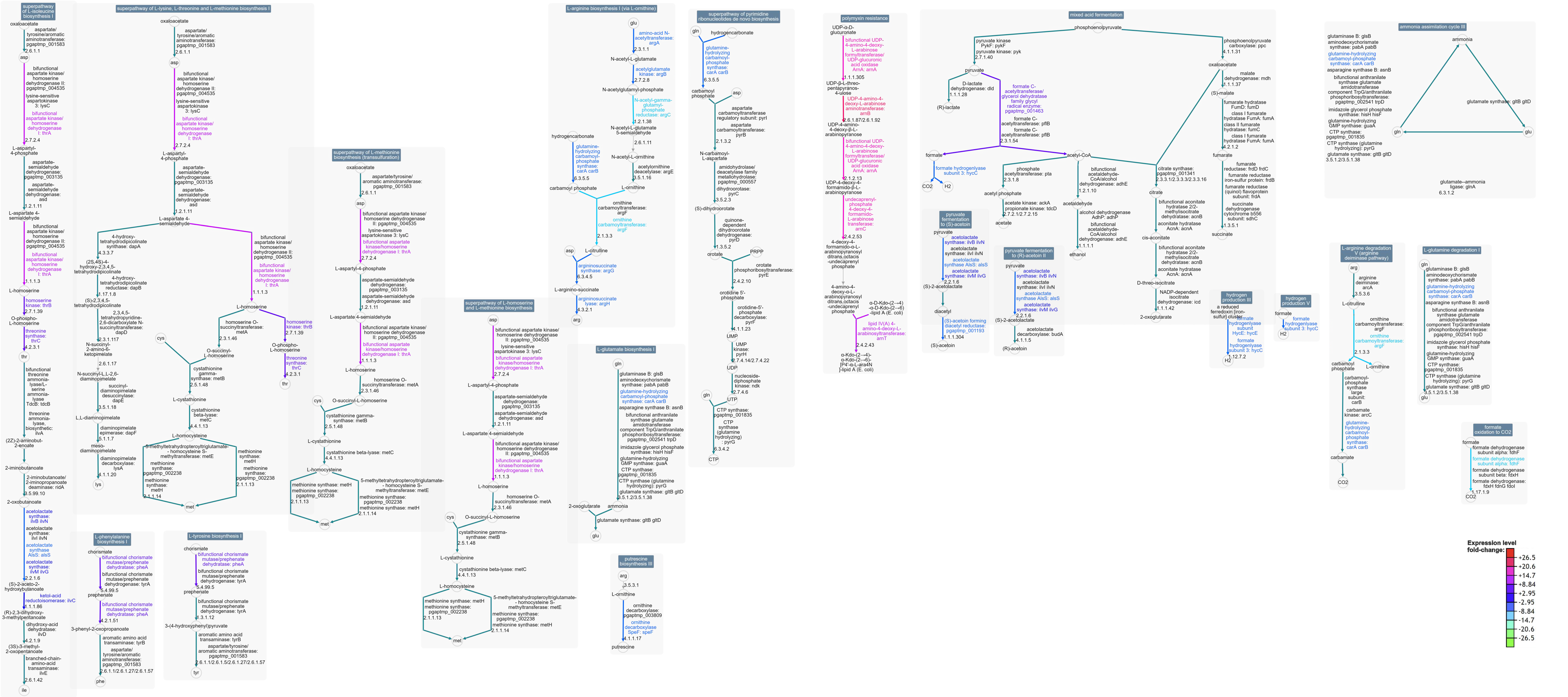

FIG S2 Metabolic pathways affected by the differentially expressed genes (DEGs) genes in Eh4236ColR isolate; the level of down- and up-regulation is shown by the heatmap from green, through blue, to red colour, respectively. The analysis and results visualization was performed with the Pathway Tools v26.0 software (16).
