# Supplementary material for: Glyoxylate Shunt and Pyruvate-to-Acetoin Shift Are Specific Stress Responses Induced by Colistin and Ceragenin CSA-13 in Enterobacter hormaechei ST89
Source: Microbiol Spectr. 2023 Jun 20;11(4):e01215-23. doi: 10.1128/spectrum.01215-23 (PMC10434160; doi:10.1128/spectrum.01215-23)
Supplement: Supplemental file 2 — Fig. S3 to S15. Download spectrum.01215-23-s0002.pdf, PDF file, 1.0 MB [file spectrum.01215-23-s0002.pdf]

Glyoxylate shunt and pyruvate to acetoin shift are specific stress responses induced by colistin and ceragenin CSA-13 in *Enterobacter hormaechei* ST89

Suhanya V. Prasad[1], Krzysztof Fiedoruk[1], Magdalena Zakrzewska[1], Paul B. Savage[2], Robert Bucki[1]

[1] Department of Medical Microbiology and Nanobiomedical Engineering, Medical University of Bialystok, Bialystok, Poland.

[2] Department of Chemistry and Biochemistry, Brigham Young University, Provo, UT 84601, USA.

Supplemental material

Figures S3 – S15

Differentially expressed genes (DEGs) representing the 'Biosynthesis' cellular function category in Eh4236ColR isolate

A)

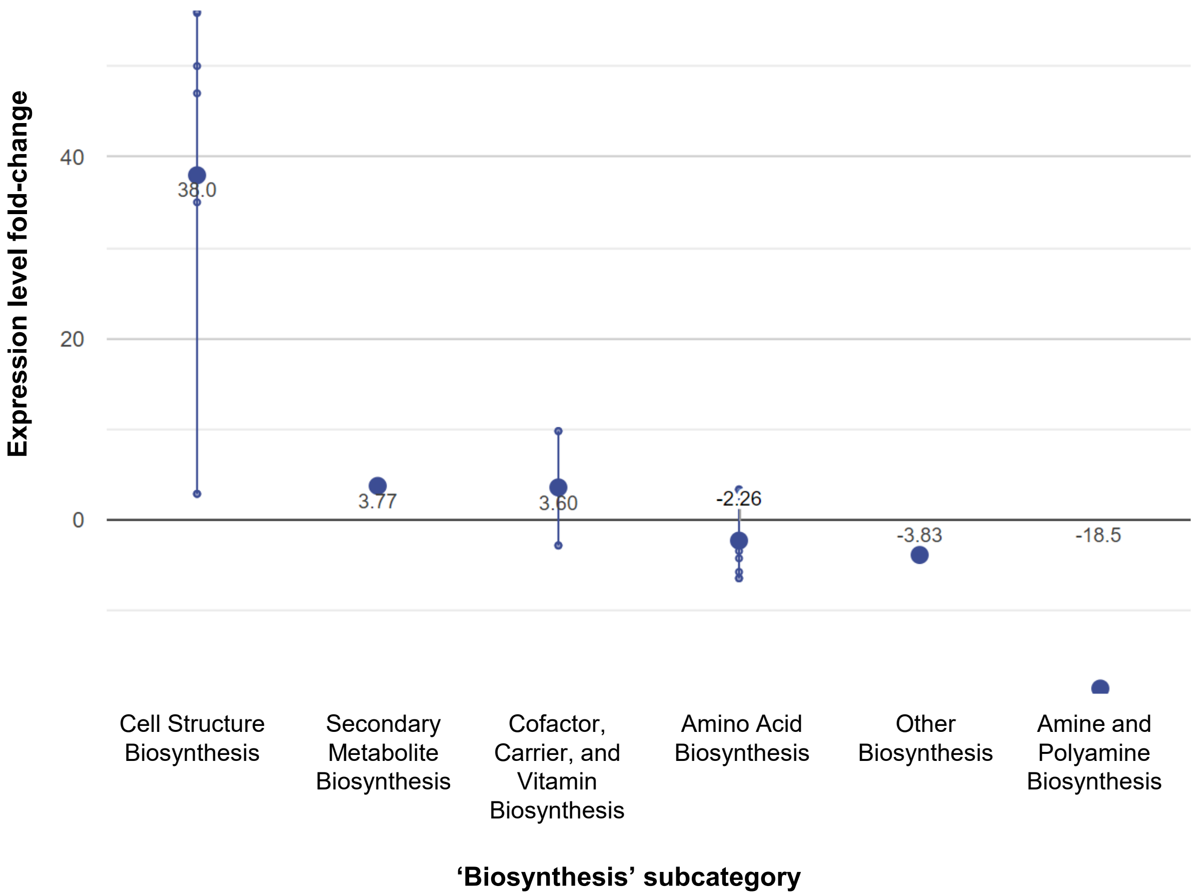

B)

'Biosynthesis' subcategory	Locus tag	Protein (gene)	Expression level fold-change
Cell Structure Biosynthesis	N5F16_22025	Udp-4-amino-4-deoxy-l-arabinose aminotransferase (arnb)	55.84
Cell Structure Biosynthesis	N5F16_22020	Undecaprenyl-phosphate 4-deoxy-4-formamido-l-arabinose transferase (arnc)	49.81
Cell Structure Biosynthesis	N5F16_22015	Bifunctional udp-4-amino-4-deoxy-l-arabinose formyltransferase/udp-glucuronic acid oxidase arna (arna)	46.81
Cell Structure Biosynthesis	N5F16_22005	Lipid IV(A) 4-amino-4-deoxy-l-arabinosyltransferase (arnt)	34.52
Cofactor, Carrier, and Vitamin Biosynthesis	N5F16_07395	Undecaprenyl-diphosphate phosphatase (ybjg)	9.81
Secondary Metabolite Biosynthesis	N5F16_21990	4'-phosphopantetheinyl transferase acpt (acpt)	3.77
Cofactor, Carrier, and Vitamin Biosynthesis	N5F16_21990	4'-phosphopantetheinyl transferase acpt (acpt)	3.77
Amino Acid Biosynthesis	N5F16_18330	Diaminopimelate decarboxylase (lysa)	3.36
Amino Acid Biosynthesis	N5F16_22515	Ketol-acid reductoisomerase (ilvc)	3.28
Cell Structure Biosynthesis	N5F16_07385	Serine-type d-ala-d-ala carboxypeptidase (dacc)	2.89
Amino Acid Biosynthesis	N5F16_20420	Argininosuccinate synthase (argg)	-2.62
Cofactor, Carrier, and Vitamin Biosynthesis	N5F16_19035	[Citrate (pro-3s)-lyase] ligase (citc)	-2.77
Amino Acid Biosynthesis	N5F16_22650	Acetylglutamate kinase (argb)	-3.43
Other Biosynthesis	N5F16_22650	Acetylglutamate kinase (argb)	-3.43
Amino Acid Biosynthesis	N5F16_22655	N-acetyl-gamma-glutamyl-phosphate reductase (argc)	-4.24
Other Biosynthesis	N5F16_22655	N-acetyl-gamma-glutamyl-phosphate reductase (argc)	-4.24
Amino Acid Biosynthesis	N5F16_20185	Bifunctional threonine ammonia-lyase/l-serine ammonia-lyase tdcb (tdcb)	-5.74
Amino Acid Biosynthesis	N5F16_02955	Ornithine carbamoyltransferase (argf)	-6.4
Amine and Polyamine Biosynthesis	N5F16_06605	Ornithine decarboxylase spef (spef)	-18.54

FIG S3 Differentially expressed genes (DEGs) representing the 'Biosynthesis' cellular function category in Eh4236ColR isolate; (panel A) graph depicting the average (the large blue dots) and individual (the large blue dots) gene expression level fold-change values for DEGs belonging to specific 'Biosynthesis' subcategories, (panel B) table with characteristics of the DEGs used to create the graph. Analysis was performed using the 'Omics' module of Pathway Tools v26.0 software (16).

Differentially expressed genes (DEGs) representing the ‘Degradation’ cellular function category in Eh4236ColR isolate

A)

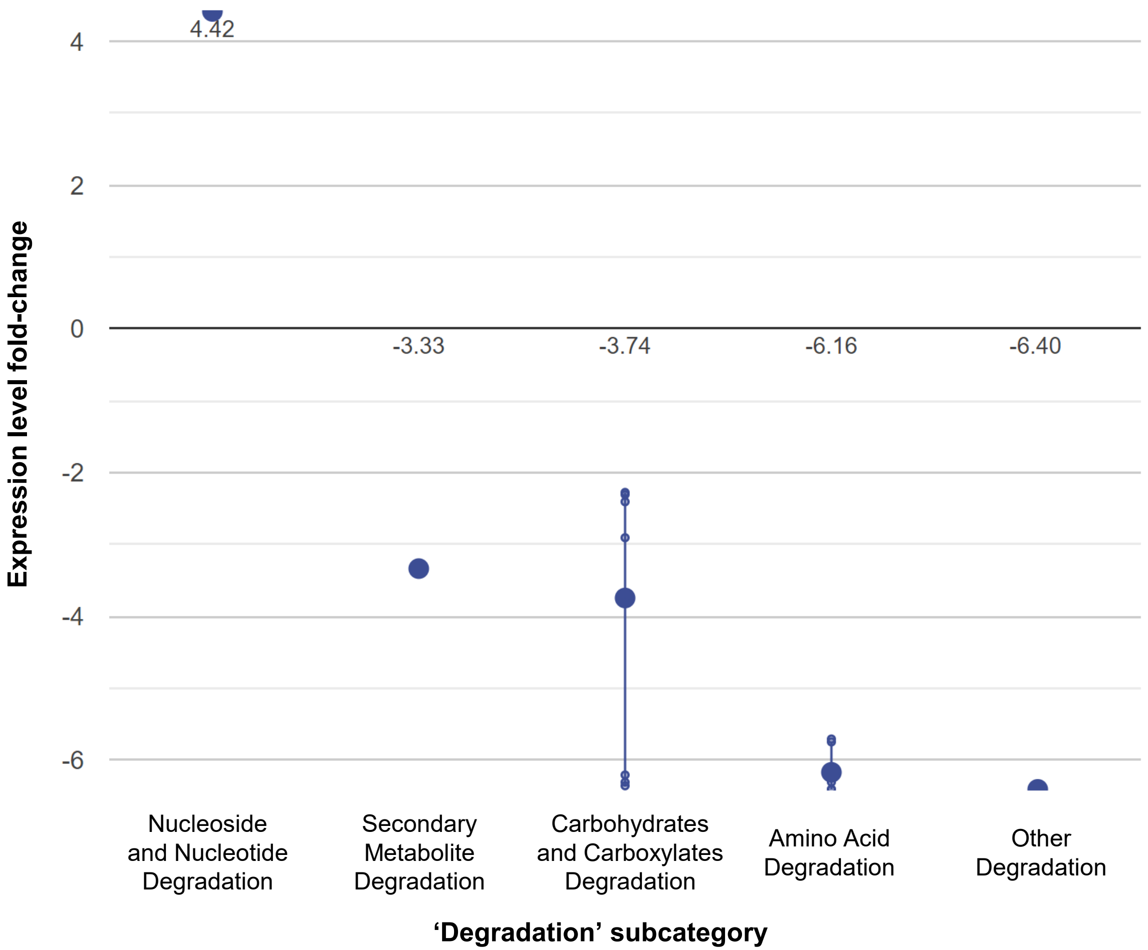

B)

‘Degradation’ subcategory	Locus tag	Protein (gene)	Expression level fold-change
Nucleoside and Nucleotide Degradation	N5F16_18130	Nucleotide 5'-monophosphate nucleosidase ppnn (ppnn)	4.42
Carbohydrates and Carboxylates Degradation	N5F16_19020	Citrate lyase subunit alpha (citf)	-2.27
Carbohydrates and Carboxylates Degradation	N5F16_19025	Citrate (pro-3s)-lyase subunit beta (cite)	-2.3
Carbohydrates and Carboxylates Degradation	N5F16_15150	Mannonate dehydratase (uxua)	-2.4
Carbohydrates and Carboxylates Degradation	N5F16_20215	Galactarate dehydratase (gard)	-2.86
Secondary Metabolite Degradation	N5F16_20050	Autoinducer-2 kinase (lsrk)	-3.33
Carbohydrates and Carboxylates Degradation	N5F16_19030	Citrate lyase acyl carrier protein (citd)	-3.78
Amino Acid Degradation	N5F16_20185	Bifunctional threonine ammonia-lyase/l-serine ammonia-lyase tdcb (tdcb)	-5.74
Carbohydrates and Carboxylates Degradation	N5F16_20170	Formate c-acetyltransferase (pflb)	-6.21
Amino Acid Degradation	N5F16_20175	Propionate kinase (tdcd)	-6.35
Carbohydrates and Carboxylates Degradation	N5F16_20175	Propionate kinase (tdcd)	-6.35
Amino Acid Degradation	N5F16_02955	Ornithine carbamoyltransferase (argf)	-6.4
Other Degradation	N5F16_02955	Ornithine carbamoyltransferase (argf)	-6.4

FIG S4 Differentially expressed genes (DEGs) representing the ‘Degradation’ cellular function category in Eh4236ColR isolate; (panel A) graph depicting the average (the large blue dots) and individual (the large blue dots) gene expression level fold-change values for DEGs belonging to specific ‘Degradation’ subcategories, (panel B) table with characteristics of the DEGs used to create the graph. Analysis was performed using the ‘Omics’ module of Pathway Tools v26.0 software (16).

Differentially expressed genes (DEGs) representing the 'Energy' cellular function category in Eh4236ColR isolate

A)

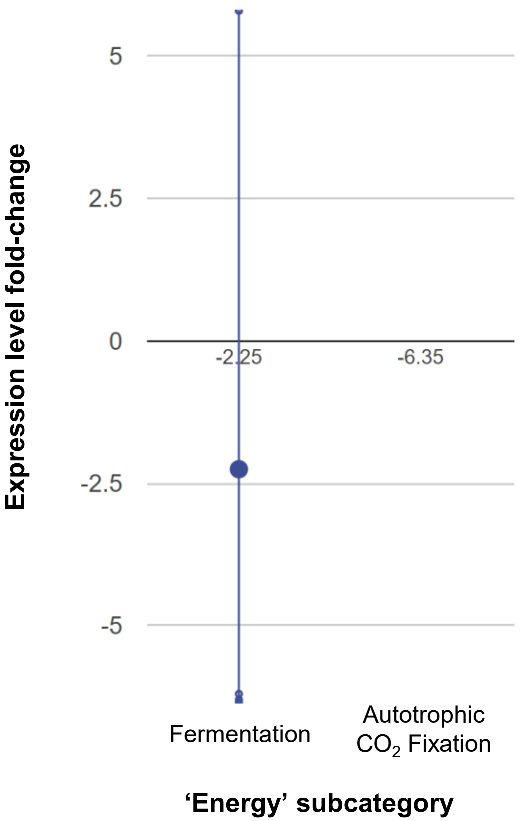

B)

'Energy' subcategory	Locus tag	Protein (gene)	Expression level fold-change
Fermentation	N5F16_05955	Acetolactate decarboxylase (buda)	5.8
Fermentation	N5F16_20170	Formate c-acetyltransferase (pflb)	-6.21
Fermentation	N5F16_20175	Propionate kinase (tdcd)	-6.35
Autotrophic CO2 Fixation	N5F16_20175	Propionate kinase (tdcd)	-6.35

FIG S5 Differentially expressed genes (DEGs) representing the 'Energy' cellular function category in Eh4236ColR isolate; (panel A) graph depicting the average (the large blue dots) and individual (the large blue dots) gene expression level fold-change values for DEGs belonging to specific Energy' subcategories, (panel B) table with characteristics of the DEGs used to create the graph. Analysis was performed using the 'Omics' module of Pathway Tools v26.0 software (16).

Differentially expressed genes (DEGs) representing the ‘Other Pathways’ category in Eh4236ColR isolate

A)

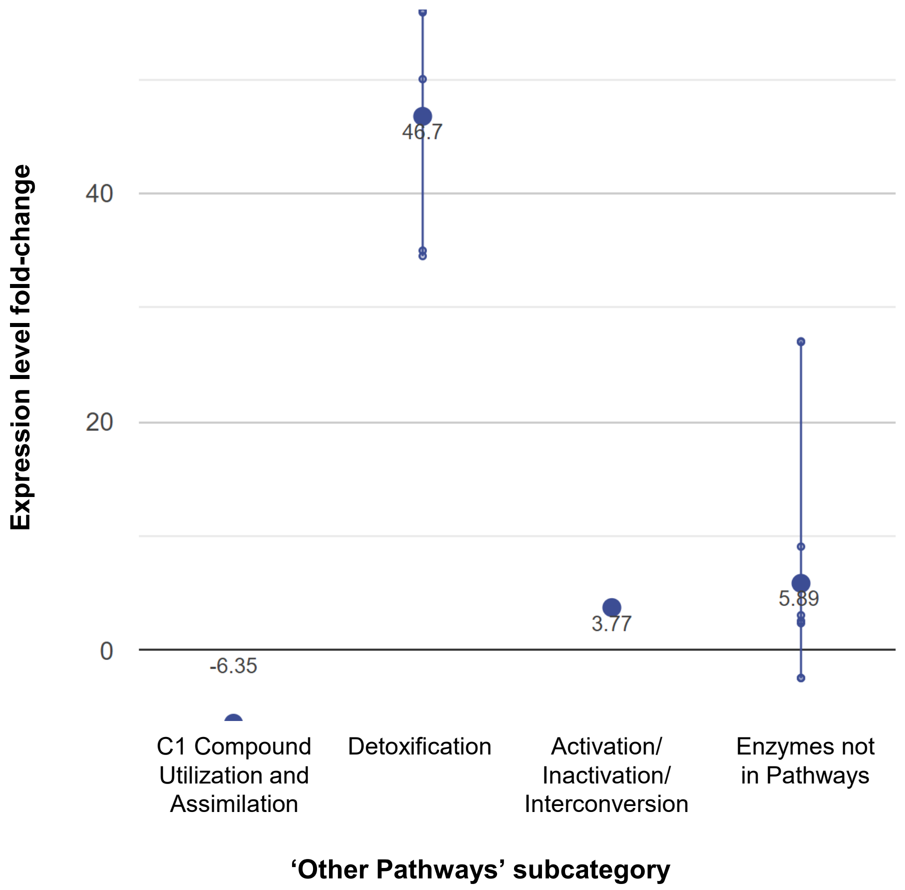

B)

‘Other Pathways’ subcategory	Locus tag	Protein (gene)	Expression level fold-change
Detoxification	N5F16_22025	Udp-4-amino-4-deoxy-l-arabinose aminotransferase (arnb)	55.84
Detoxification	N5F16_22020	Undecaprenyl-phosphate 4-deoxy-4-formamido-l-arabinose transferase (arnc)	49.81
Detoxification	N5F16_22015	Bifunctional udp-4-amino-4-deoxy-l-arabinose formyltransferase/udp-glucuronic acid oxidase arna (arna)	46.81
Detoxification	N5F16_22005	Lipid IV(A) 4-amino-4-deoxy-l-arabinosyltransferase (arnt)	34.52
Enzymes not in Pathways	N5F16_06330	Lipid IV(A) palmitoyltransferase pagp (pagp)	27.06
Enzymes not in Pathways	N5F16_14385	Lipid IV(A) palmitoyltransferase pagp (pagp)	9.07
Activation/Inactivation/Interconversion	N5F16_21990	4'-phosphopantetheinyl transferase acpt (acpt)	3.77
Enzymes not in Pathways	N5F16_10855	Spermidine n1-acetyltransferase (speg)	3.14
Enzymes not in Pathways	N5F16_07220	Aldo/keto reductase (N5F16_07220)	2.72
Enzymes not in Pathways	N5F16_19105	Poly-beta-1,6-n-acetyl-d-glucosamine n-deacetylase pgab (pgab)	2.65
Enzymes not in Pathways	N5F16_22030	Phenolic acid decarboxylase (N5F16_22030)	2.5
Enzymes not in Pathways	N5F16_19110	Poly-beta-1,6-n-acetyl-d-glucosamine synthase (pgac)	2.38
Enzymes not in Pathways	N5F16_16045	Beta-galactosidase subunit alpha (ebga)	-2.39
C1 Compound Utilization and Assimilation	N5F16_20175	Propionate kinase (tdcd)	-6.35

FIG S6 Differentially expressed genes (DEGs) representing the ‘Other Pathways’ category in Eh4236ColR isolate; (panel A) graph depicting the average (the large blue dots) and individual (the large blue dots) gene expression level fold-change values for DEGs belonging to specific ‘Other Pathways’ subcategories, (panel B) table with characteristics of the DEGs used to create the graph. Analysis was performed using the ‘Omics’ module of Pathway Tools v26.0 software (16).

Differentially expressed genes (DEGs) representing the 'Cell Exterior' category in Eh4236ColR isolate

A)

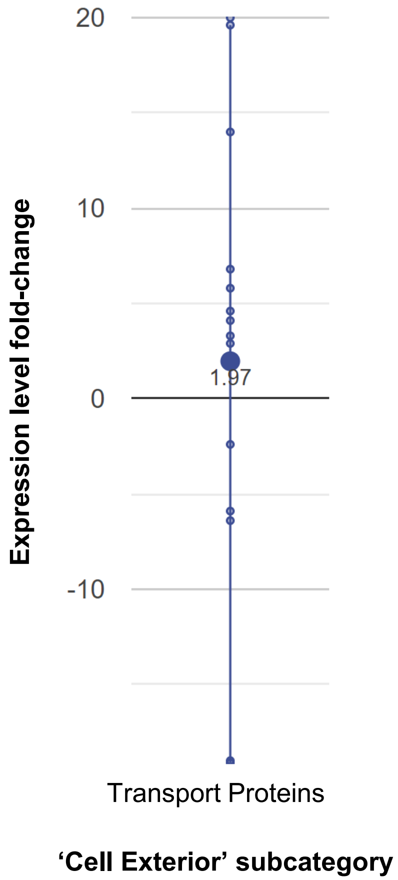

B)

'Cell Exterior' subcategory	Locus tag	Protein (gene)	Expression level fold-change
Transport Proteins	N5F16_08425-MONOMER	Magnesium transporter (08425)	19.61
Transport Proteins	CPLX2OCP-28	4-amino-4-deoxy-l-arabinose-phosphoundecaprenol flippase	14.47
Transport Proteins	N5F16_02860-MONOMER	Magnesium-translocating p-type atpase (02860)	6.75
Transport Proteins	CPLX2OCP-29	Potassium-transporting atpase	5.85
Transport Proteins	N5F16_09990-MONOMER	Outer membrane lipoprotein slyb (09990)	4.63
Transport Proteins	N5F16_12955-MONOMER	Nark family nitrate/nitrite MFS transporter (12955)	4.07
Transport Proteins	N5F16_15265-MONOMER	Magnesium transporter (15265)	3.28
Transport Proteins	N5F16_19100-MONOMER	Poly-beta-1,6 n-acetyl-d-glucosamine export porin pgaa (19100)	2.93
Transport Proteins	N5F16_15340-MONOMER	Glycerol-3-phosphate transporter (15340)	-2.33
Transport Proteins	CPLX2OCP-33	PTS glucitol/sorbitol transporter	-2.37
Transport Proteins	N5F16_20180-MONOMER	Threonine/serine transporter tdcc (20180)	-5.86
Transport Proteins	CPLX2OCP-16	Putative autoinducer 2 ABC transporter	-6.36
Transport Proteins	N5F16_06600-MONOMER	Putrescine-ornithine antiporter (06600)	-19.12

FIG S7 Differentially expressed genes (DEGs) representing the 'Cell Exterior' category in Eh4236ColR isolate; (panel A) graph depicting the average (the large blue dots) and individual (the large blue dots) gene expression level fold-change values for DEGs belonging to specific 'Cell Exterior' subcategories, (panel B) table with characteristics of the DEGs used to create the graph. Analysis was performed using the 'Omics' module of Pathway Tools v26.0 software (16).

Differentially expressed genes (DEGs) representing the ‘Objects Not Present in any Subsystem’ category in Eh4236ColR isolate

A)

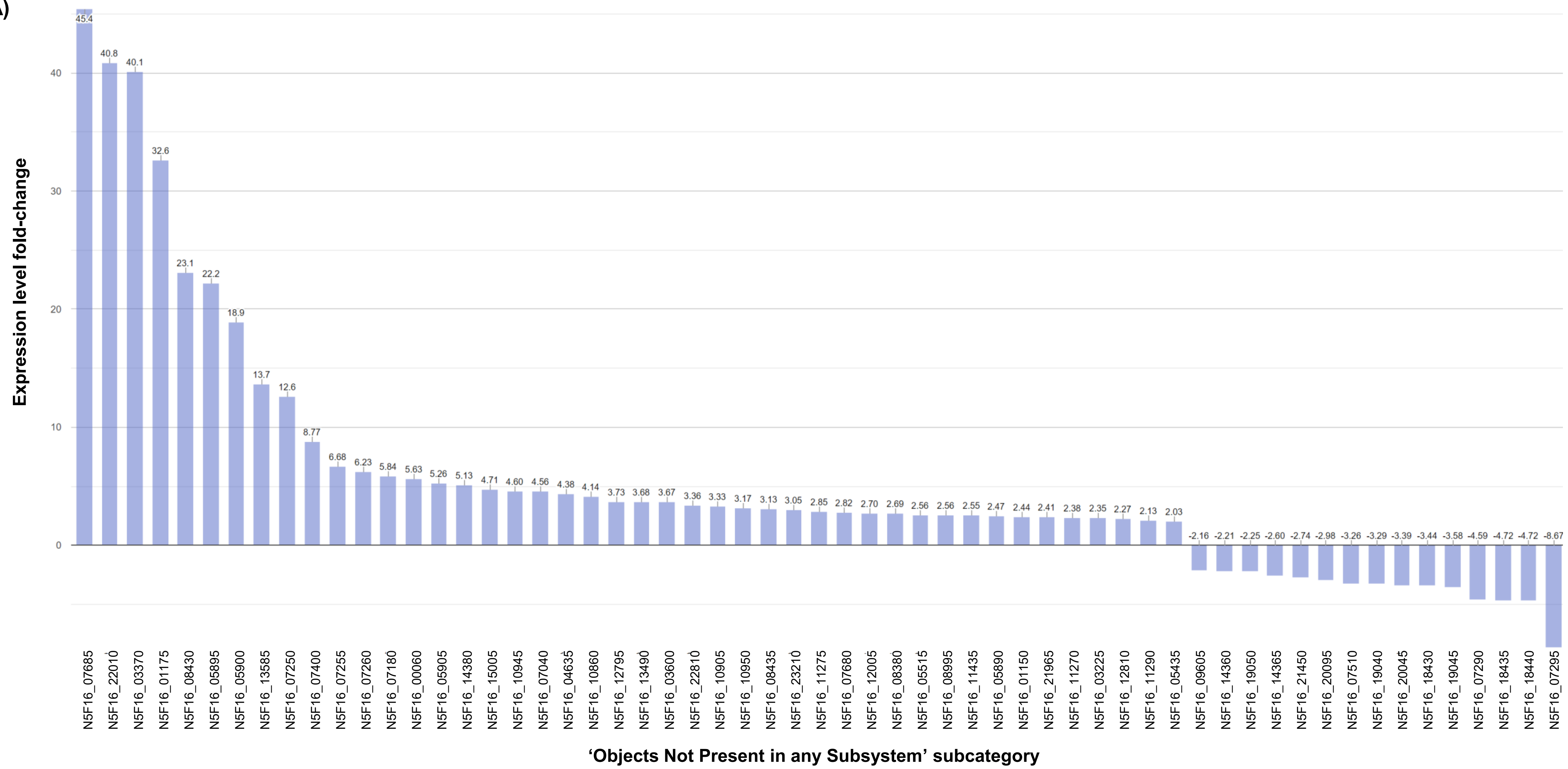

FIG S8 Differentially expressed genes (DEGs) representing the ‘Objects Not Present in any Subsystem’ category in Eh4236ColR isolate; (panel A) graph depicting the average (the large blue dots) and individual (the large blue dots) gene expression level fold-change values for DEGs belonging to specific ‘Objects Not Present in any Subsystem’ subcategories, (panel B, next page) table with characteristics of the DEGs used to create the graph. Analysis was performed using the ‘Omics’ module of Pathway Tools v26.0 software (16).

Differentially expressed genes (DEGs) representing the ‘Objects Not Present in any Subsystem’ category in Eh4236ColR isolate, cont.

B)

Locus tag	Protein (gene)	Expression level fold-change
N5F16_07685	Virk/ybjx family protein	45.44
N5F16_22010	4-deoxy-4-formamido-l-arabinose- phosphoundecaprenol deformylase (arnd)	40.84
N5F16_03370	Yfaz family protein	40.15
N5F16_01175	MFS transporter	32.61
N5F16_08430	Deda family protein	23.14
N5F16_05895	Cation-transporting p-type atpase	22.24
N5F16_05900	Efflux RND transporter periplasmic adaptor subunit	18.93
N5F16_13585	Virk/ybjx family protein	13.65
N5F16_07250	Efflux RND transporter periplasmic adaptor subunit	12.6
N5F16_07400	Phosphatase PAP2 family protein	8.77
N5F16_07255	Macb family efflux pump subunit	6.68
N5F16_07260	Efflux transporter outer membrane subunit	6.23
N5F16_07180	Outer membrane protein ompx (ompx)	5.84
N5F16_00060	MFS transporter	5.63
N5F16_05905	Efflux RND transporter permease subunit	5.26
N5F16_14380	Acyltransferase family protein	5.13
N5F16_15005	Deda family protein	4.71
N5F16_10945	Extracellular solute-binding protein	4.6
N5F16_07040	Bax inhibitor-1/ycca family protein	4.56
N5F16_04635	Envelope stress response activation lipoprotein nlpe (nlpe)	4.38
N5F16_10860	DUF1283 family protein	4.14
N5F16_12795	Dna-binding transcriptional regulator	3.73
N5F16_13490	Manganese efflux pump mntp (mntp)	3.68
N5F16_03600	Patatin family protein	3.67
N5F16_22810	Cell-envelope stress modulator cpxp (cpxp)	3.36
N5F16_10905	Universal stress protein	3.33
N5F16_10950	ABC transporter permease subunit	3.17
N5F16_08435	SDR family oxidoreductase	3.13
N5F16_23210	Transcriptional regulator asnc (asnc)	3.05
N5F16_11275	Type I secretion system permease/atpase	2.85
N5F16_07680	Atp-dependent endonuclease	2.82
N5F16_12005	Elongation factor G (fusa))	2.7
N5F16_08380	Ftsh protease modulator ycca (ycca)	2.69
N5F16_05515	Hha toxicity modulator tomb (tomb)	2.56
N5F16_08995	Glycine zipper 2TM domain-containing protein	2.56
N5F16_11435	Yncj family protein	2.55
N5F16_05890	DUF1158 domain-containing protein	2.47
N5F16_01150	Ompa family lipoprotein	2.44
N5F16_21965	DUF3261 domain-containing protein	2.41
N5F16_11270	Ig-like domain-containing protein	2.38
N5F16_03225	Amidohydrolase	2.35
N5F16_12810	Phage virion morphogenesis protein	2.27
N5F16_11290	EAL domain-containing protein	2.13
N5F16_05435	Hypothetical protein	2.03
N5F16_09605	MFS transporter	-2.16
N5F16_14360	Cation-transporting p-type atpase	-2.21
N5F16_19050	Sensor histidine kinase	-2.25
N5F16_14365	Universal stress protein	-2.6
N5F16_21450	Aspartate aminotransferase family protein	-2.74
N5F16_20095	MFS transporter	-2.98
N5F16_07510	ABC transporter substrate-binding protein artj (artj)	-3.26
N5F16_19040	Fumarylacetoacetate hydrolase family protein	-3.29
N5F16_20045	Transcriptional regulator lsrr (lsrr)	-3.39
N5F16_18430	General stress protein	-3.44
N5F16_19045	2-hydroxycarboxylate transporter family protein	-3.58
N5F16_07290	Glycoside hydrolase family 31 protein	-4.59
N5F16_18435	Ferritin-like domain-containing protein	-4.72
N5F16_18440	Ferritin-like domain-containing protein	-4.72
N5F16_07295	MFS transporter	-8.67

FIG S8 cont. Differentially expressed genes (DEGs) representing the ‘Objects Not Present in any Subsystem’ category in Eh4236ColR isolate; (panel A, previous page) graph depicting the average (the large blue dots) and individual (the large blue dots) gene expression level fold-change values for DEGs belonging to specific ‘Objects Not Present in any Subsystem’ subcategories, (panel B) table with characteristics of the DEGs used to create the graph. Analysis was performed using the ‘Omics’ module of Pathway Tools v26.0 software (16).

Differentially expressed genes (DEGs) representing the 'Biosynthesis' cellular function category in Eh4236Csa13R isolate

A)

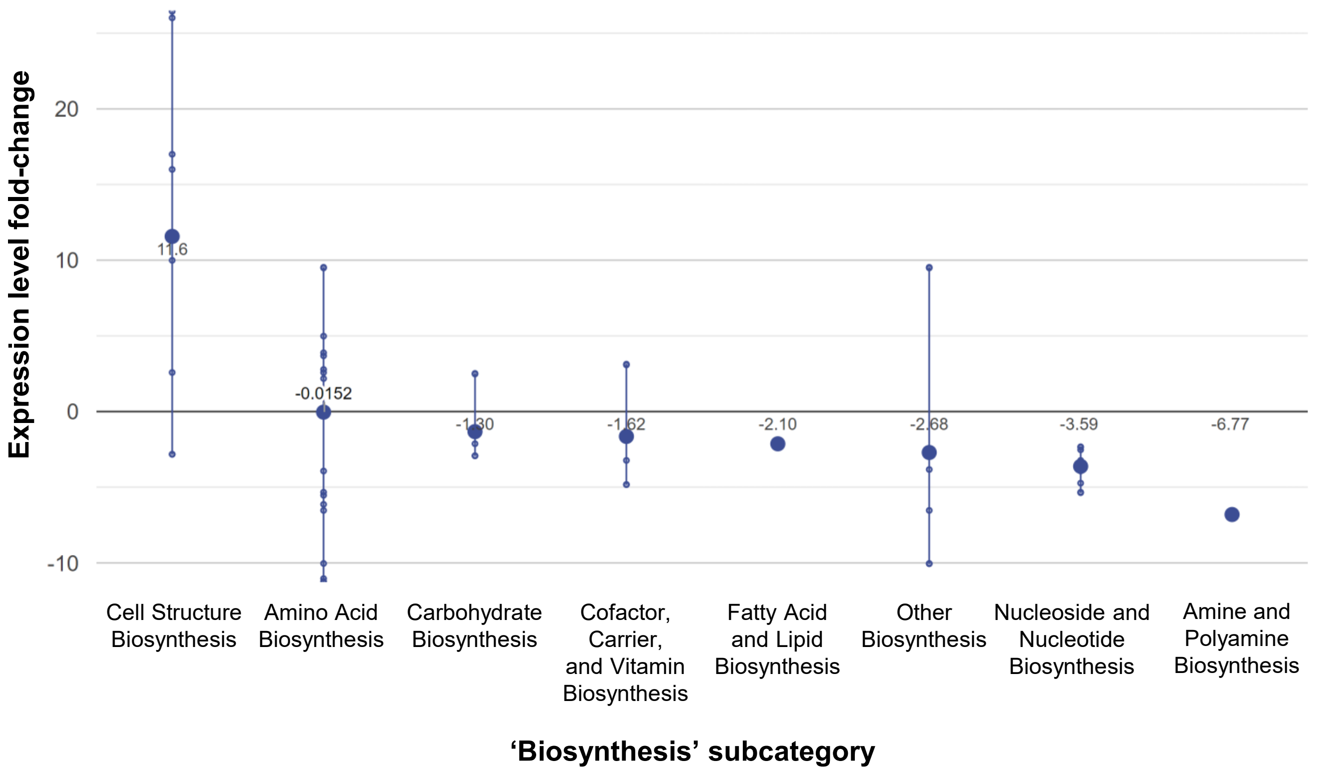

B)

'Biosynthesis' subcategory	Locus tag	Protein (gene)	Expression level fold-change
Cell Structure Biosynthesis	N5F16_22025	UDP-4-amino-4-deoxy-L-arabinose aminotransferase (arnB)	26.43
Cell Structure Biosynthesis	N5F16_22020	undecaprenyl-phosphate 4-deoxy-4-formamido-L-arabinose transferase (arnC)	17.14
Cell Structure Biosynthesis	N5F16_22015	bifunctional UDP-4-amino-4-deoxy-L-arabinose formyltransferase/UDP-glucuronic acid oxidase ArnA (arnA)	15.66
Cell Structure Biosynthesis	N5F16_22005	lipid IV(A) 4-amino-4-deoxy-L-arabinosyltransferase (arnT)	10.39
Amino Acid Biosynthesis	N5F16_03730	bifunctional aspartate kinase/homoserine dehydrogenase I (thrA)	9.54
Other Biosynthesis	N5F16_03730	bifunctional aspartate kinase/homoserine dehydrogenase I (thrA)	9.54
Amino Acid Biosynthesis	N5F16_16745	bifunctional chorismate mutase/prephenate dehydratase (pheA)	5.05
Amino Acid Biosynthesis	N5F16_04035	3-isopropylmalate dehydrogenase (leuB)	5
Amino Acid Biosynthesis	N5F16_04030	3-isopropylmalate dehydratase large subunit (leuC)	3.9
Amino Acid Biosynthesis	N5F16_03740	threonine synthase (thrC)	3.8
Amino Acid Biosynthesis	N5F16_03735	homoserine kinase (thrB)	3.77
Amino Acid Biosynthesis	N5F16_04025	3-isopropylmalate dehydratase small subunit (leuD)	3.76
Amino Acid Biosynthesis	N5F16_04040	2-isopropylmalate synthase (leuA)	3.71
Cofactor, Carrier, and Vitamin Biosynthesis	N5F16_07395	undecaprenyl-diphosphate phosphatase (ybjG)	3.15
Amino Acid Biosynthesis	N5F16_22515	ketol-acid reductoisomerase (ilvC)	2.89
Amino Acid Biosynthesis	N5F16_22540	acetolactate synthase 2 small subunit (ilvM)	2.84
Amino Acid Biosynthesis	N5F16_00165	acetolactate synthase large subunit (ilvB)	2.83
Cell Structure Biosynthesis	N5F16_07385	serine-type D-Ala-D-Ala carboxypeptidase (dacC)	2.65
Amino Acid Biosynthesis	N5F16_00170	acetolactate synthase small subunit (ilvN)	2.61
Carbohydrate Biosynthesis	N5F16_06895	UDP-glucose 4-epimerase GalE (galE)	2.54
Amino Acid Biosynthesis	N5F16_22545	acetolactate synthase 2 catalytic subunit (ilvG)	2.17
Carbohydrate Biosynthesis	N5F16_18775	class II fructose-bisphosphatase (glpX)	-2.06
Fatty Acid and Lipid Biosynthesis	N5F16_09880	cyclopropane fatty acyl phospholipid synthase (cfa)	-2.1
Fatty Acid and Lipid Biosynthesis	N5F16_20865	acetyl-CoA carboxylase biotin carboxylase subunit (accC)	-2.11
Nucleoside and Nucleotide Biosynthesis	N5F16_01370	bifunctional phosphoribosylaminoimidazolecarboxamide formyltransferase/IMP cyclohydrolase (purH)	-2.33
Nucleoside and Nucleotide Biosynthesis	N5F16_15320	ribonucleoside-diphosphate reductase subunit alpha (nrdA)	-2.46
Carbohydrate Biosynthesis	N5F16_22190	UDP-forming cellulose synthase catalytic subunit (bcsA)	-2.79
Cell Structure Biosynthesis	N5F16_22190	UDP-forming cellulose synthase catalytic subunit (bcsA)	-2.79
Carbohydrate Biosynthesis	N5F16_23280	glutamine--fructose-6-phosphate transaminase (isomerizing) (glmS)	-2.89
Nucleoside and Nucleotide Biosynthesis	N5F16_05690	5-(carboxyamino)imidazole ribonucleotide synthase (purK)	-3.2
Nucleoside and Nucleotide Biosynthesis	N5F16_16215	phosphoribosylglycinamide formyltransferase (purN)	-3.21
Cofactor, Carrier, and Vitamin Biosynthesis	N5F16_16215	phosphoribosylglycinamide formyltransferase (purN)	-3.21
Amino Acid Biosynthesis	N5F16_18210	amino-acid N-acetyltransferase (argA)	-3.78
Other Biosynthesis	N5F16_18210	amino-acid N-acetyltransferase (argA)	-3.78
Amino Acid Biosynthesis	N5F16_20420	argininosuccinate synthase (argG)	-3.87
Nucleoside and Nucleotide Biosynthesis	N5F16_05695	5-(carboxyamino)imidazole ribonucleotide mutase (purE)	-3.89
Nucleoside and Nucleotide Biosynthesis	N5F16_16210	phosphoribosylformylglycinamidine cyclo-ligase (purM)	-4.71
Cofactor, Carrier, and Vitamin Biosynthesis	N5F16_17860	hydrogenase formation protein HypD (hypD)	-4.79
Amino Acid Biosynthesis	N5F16_03880	glutamine-hydrolyzing carbamoyl-phosphate synthase small subunit (carA)	-5.33
Nucleoside and Nucleotide Biosynthesis	N5F16_03880	glutamine-hydrolyzing carbamoyl-phosphate synthase small subunit (carA)	-5.33
Amino Acid Biosynthesis	N5F16_05960	acetolactate synthase AlsS (alsS)	-5.48
Amino Acid Biosynthesis	N5F16_22645	argininosuccinate lyase (argH)	-6.08
Amino Acid Biosynthesis	N5F16_22650	acetylglutamate kinase (argB)	-6.45
Other Biosynthesis	N5F16_22650	acetylglutamate kinase (argB)	-6.45
Amine and Polyamine Biosynthesis	N5F16_06605	ornithine decarboxylase SpeF (speF)	-6.77
Amino Acid Biosynthesis	N5F16_22655	N-acetyl-gamma-glutamyl-phosphate reductase (argC)	-10.02
Other Biosynthesis	N5F16_22655	N-acetyl-gamma-glutamyl-phosphate reductase (argC)	-10.02
Amino Acid Biosynthesis	N5F16_02955	ornithine carbamoyltransferase (argF)	-11.18

FIG S9 Differentially expressed genes (DEGs) representing the 'Biosynthesis' cellular function category in Eh4236Csa13R isolate; (panel A) graph depicting the average (the large blue dots) and individual (the large blue dots) gene expression level fold-change values for DEGs belonging to specific 'Biosynthesis' subcategory, (panel B) table with characteristics of the DEGs used to create the graph. Analysis was performed using the 'Omics' module of Pathway Tools v26.0 software (16).

Differentially expressed genes (DEGs) representing the 'Degradation' cellular function category in Eh4236Csa13R isolate

A)

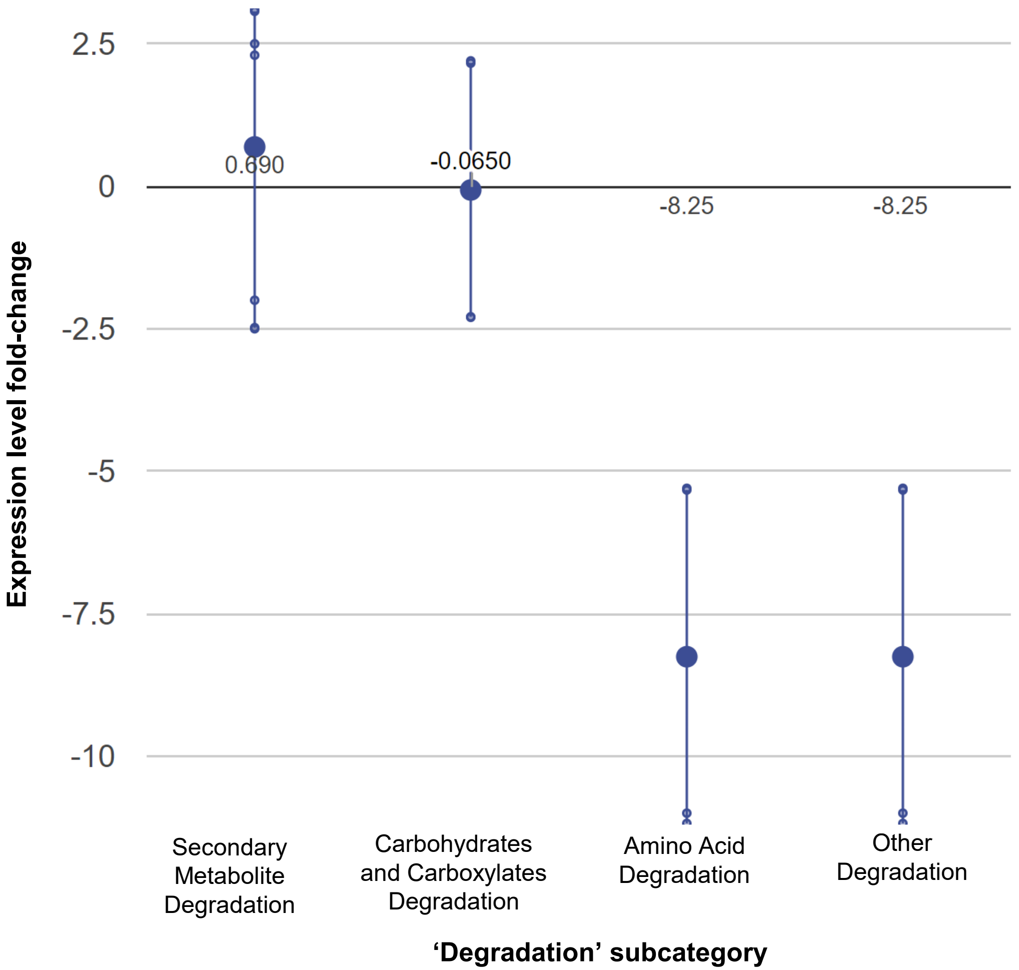

B)

'Degradation' subcategory	Locus tag	Protein (gene)	Expression level fold-change
Amino Acid Degradation	N5F16_02955	Ornithine carbamoyltransferase (argf)	-11.18
Other Degradation	N5F16_02955	Ornithine carbamoyltransferase (argf)	-11.18
Amino Acid Degradation	N5F16_03880	Glutamine-hydrolyzing carbamoyl-phosphate synthase small subunit (cara)	-5.33
Other Degradation	N5F16_03880	Glutamine-hydrolyzing carbamoyl-phosphate synthase small subunit (cara)	-5.33
Carbohydrates and Carboxylates Degradation	N5F16_23185	D-ribose pyranase (rbsd)	-2.48
Secondary Metabolite Degradation	N5F16_17735	Sorbitol-6-phosphate dehydrogenase (srld)	-2.29
Carbohydrates and Carboxylates Degradation	N5F16_13645	Phosphogluconate dehydratase (edd)	-2.01
Secondary Metabolite Degradation	N5F16_16555	N-acetylmuramic acid 6-phosphate etherase (murq)	2.16
Carbohydrates and Carboxylates Degradation	N5F16_21655	Maltodextrin phosphorylase (malp)	2.33
Carbohydrates and Carboxylates Degradation	N5F16_06895	Udp-glucose 4-epimerase gale (gale)	2.54
Carbohydrates and Carboxylates Degradation	N5F16_07315	Formate c-acetyltransferase/glycerol dehydratase family glycyl radical enzyme	3.07

Supplementary Figure S10. Differentially expressed genes (DEGs) representing the 'Degradation' cellular function category in Eh4236Csa13R isolate; (panel A) graph depicting the average (the large blue dots) and individual (the large blue dots) gene expression level fold-change values for DEGs belonging to specific 'Degradation' subcategory, (panel B) table with characteristics of the DEGs used to create the graph. Analysis was performed using the 'Omics' module of Pathway Tools v26.0 software (16).

Differentially expressed genes (DEGs) representing the 'Energy' cellular function category in Eh4236Csa13R isolate

A)

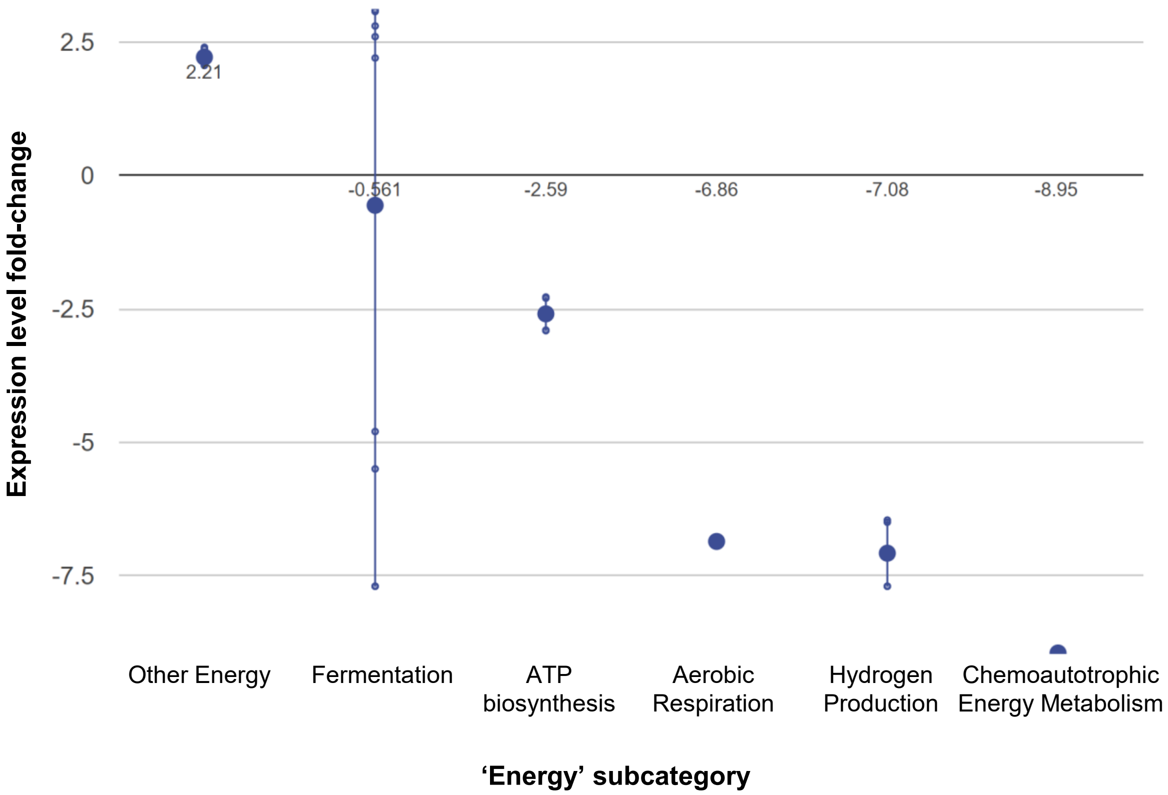

B)

'Energy' subcategory	Locus tag	Protein (gene)	Expression level fold-change
Fermentation	N5F16_07315	Formate c-acetyltransferase/glycerol dehydratase family glycyl radical enzyme	3.07
Fermentation	N5F16_22540	Acetolactate synthase 2 small subunit (ilvm)	2.84
Fermentation	N5F16_00165	Acetolactate synthase large subunit (ilvb)	2.83
Fermentation	N5F16_00170	Acetolactate synthase small subunit (ilvn)	2.61
Other Energy	N5F16_01395	Malate synthase A (aceb)	2.37
Fermentation	N5F16_22545	Acetolactate synthase 2 catalytic subunit (ilvg)	2.17
Other Energy	N5F16_01400	Isocitrate lyase (acea)	2.06
ATP biosynthesis	N5F16_23270	F0F1 ATP synthase subunit epsilon	-2.28
ATP biosynthesis	N5F16_23260	F0F1 ATP synthase subunit gamma (atpg)	-2.91
Fermentation	N5F16_05965	(S)-acetoin forming diacetyl reductase	-4.83
Fermentation	N5F16_05960	Acetolactate synthase alss (alss)	-5.48
Hydrogen Production	N5F16_17820	Formate hydrogenlyase subunit hyce (hyce)	-6.46
Aerobic Respiration	N5F16_06775	Cytochrome bd-i oxidase subunit cydx (cydx)	-6.86
Fermentation	N5F16_17830	Formate hydrogenlyase subunit 3 (hycc)	-7.7
Chemoautotrophic Energy Metabolism	N5F16_09680	formate dehydrogenase subunit alpha (fdhF)	-8.95
Other Energy	N5F16_01395	malate synthase A (aceB)	2.37
Other Energy	N5F16_01400	isocitrate lyase (aceA)	2.06

FIG S11 Differentially expressed genes (DEGs) representing the 'Energy' cellular function category in Eh4236Csa13R isolate; (panel A) graph depicting the average (the large blue dots) and individual (the large blue dots) gene expression level fold-change values for DEGs belonging to 'Energy' subcategories, (panel B) table with characteristics of the DEGs used to create the graph. Analysis was performed using the 'Omics module of Pathway Tools v26.0 software (16).

Differentially expressed genes (DEGs) representing the 'Other Pathways' category in Eh4236Csa13R isolate

A)

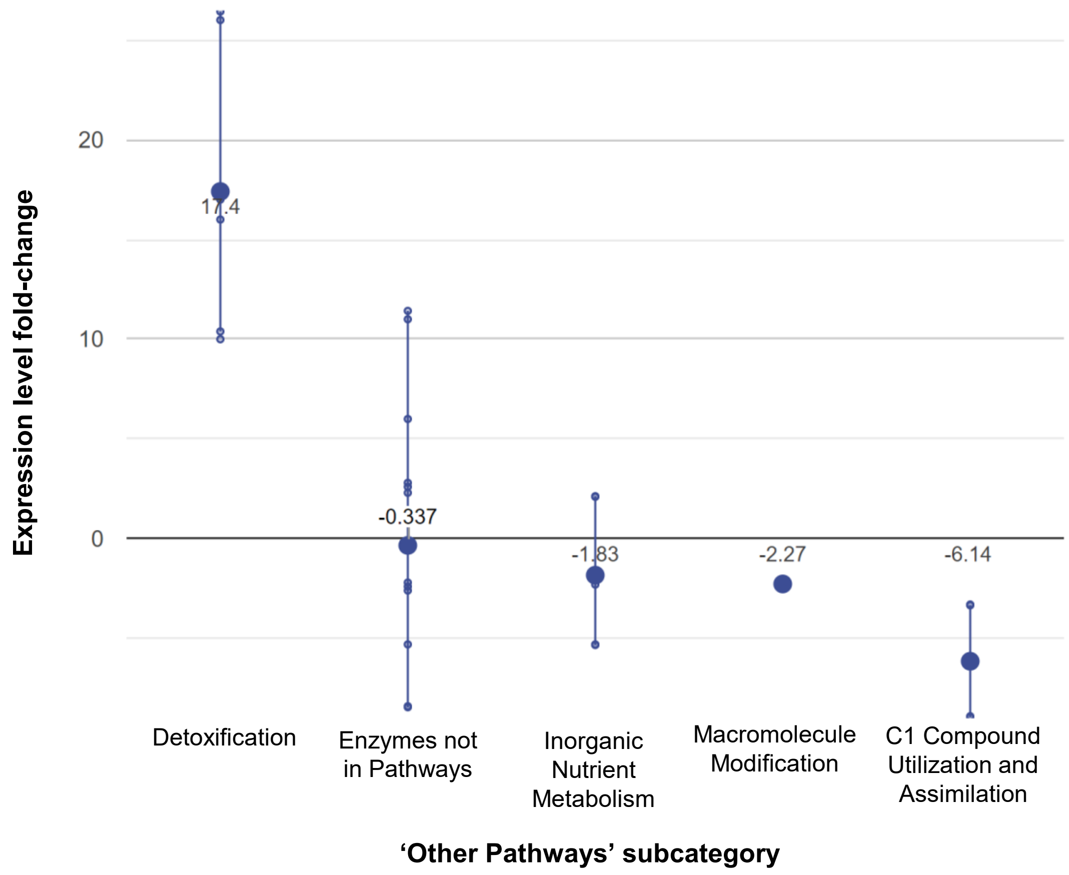

B)

'Other Pathways' subcategory	Locus tag	Protein (gene)	Expression level fold-change
Detoxification	N5F16_22025	Udp-4-amino-4-deoxy-l-arabinose aminotransferase (arnb)	26.43
Detoxification	N5F16_22020	Undecaprenyl-phosphate 4-deoxy-4-formamido-l-arabinose transferase (arnc)	17.14
Detoxification	N5F16_22015	Bifunctional udp-4-amino-4-deoxy-l-arabinose formyltransferase/udp-glucuronic acid oxidase arna (arna)	15.66
Enzymes not in Pathways	N5F16_06330	Lipid IV(A) palmitoyltransferase pagp (pagp)	11.42
Detoxification	N5F16_22005	Lipid IV(A) 4-amino-4-deoxy-l-arabinosyltransferase (arnt)	10.39
Enzymes not in Pathways	N5F16_14385	Lipid IV(A) palmitoyltransferase pagp (pagp)	5.96
Enzymes not in Pathways	N5F16_16180	N(4)-(beta-n-acetylglucosaminyl)-l-asparaginase	2.83
Enzymes not in Pathways	N5F16_07220	Aldo/keto reductase	2.64
Enzymes not in Pathways	N5F16_13795	Chemotaxis response regulator protein-glutamate methylesterase	2.43
Enzymes not in Pathways	N5F16_13785	Protein phosphatase chez (chez)	2.25
Inorganic Nutrient Metabolism	N5F16_02095	Ribose 1,5-bisphosphokinase (phnn)	2.13
Enzymes not in Pathways	N5F16_13050	Peptide chain release factor n(5)-glutamine methyltransferase (prmc)	-2.07
Enzymes not in Pathways	N5F16_15285	Fad:protein FMN transferase apbe (apbe)	-2.13
Enzymes not in Pathways	N5F16_16800	Trna (guanosine(37)-n1)-methyltransferase trmd (trmd)	-2.21
Macromolecule Modification	N5F16_05685	Trna 2-selenouridine(34) synthase mnmh (mnmh)	-2.27
Inorganic Nutrient Metabolism	N5F16_06225	Phosphoadenosine phosphosulfate reductase (N5F16_06225)	-2.29
Enzymes not in Pathways	N5F16_22175	Cellulose synthase complex outer membrane protein bcsc (bcsc)	-2.37
Enzymes not in Pathways	N5F16_08730	Cytochrome b (N5F16_08730)	-2.51
Enzymes not in Pathways	N5F16_20365	DEAD/DEAH family atp-dependent RNA helicase	-2.61
Enzymes not in Pathways	N5F16_21340	Fkbp-type peptidyl-prolyl cis-trans isomerase (fkpa)	-2.62
Enzymes not in Pathways	N5F16_01180	Kdo(2)-lipid A phosphoethanolamine 7''-transferase (eptb)	-2.63
C1 Compound Utilization and Assimilation	N5F16_17870	Formate hydrogenlyase transcriptional activator flha (flha)	-3.34
Enzymes not in Pathways	N5F16_17765	Nadh:flavorubredoxin reductase norw (norw)	-5.32
Inorganic Nutrient Metabolism	N5F16_03880	Glutamine-hydrolyzing carbamoyl-phosphate synthase small subunit (cara)	-5.33
Enzymes not in Pathways	N5F16_03390	Gtpase (yjia)	-8.45
C1 Compound Utilization and Assimilation	N5F16_09680	Formate dehydrogenase subunit alpha (fdhf)	-8.95

FIG S12 Differentially expressed genes (DEGs) representing the 'Other Pathways' category in Eh4236Csa13R isolate; (panel A) graph depicting the average (the large blue dots) and individual (the large blue dots) gene expression level fold-change values for DEGs belonging to specific 'Other Pathways' subcategories, (panel B) table with characteristics of the DEGs used to create the graph. Analysis was performed using the 'Omics' module of Pathway Tools v26.0 software (16).

Differentially expressed genes (DEGs) representing the 'Cell Exterior' category in Eh4236Csa13R isolate

A)

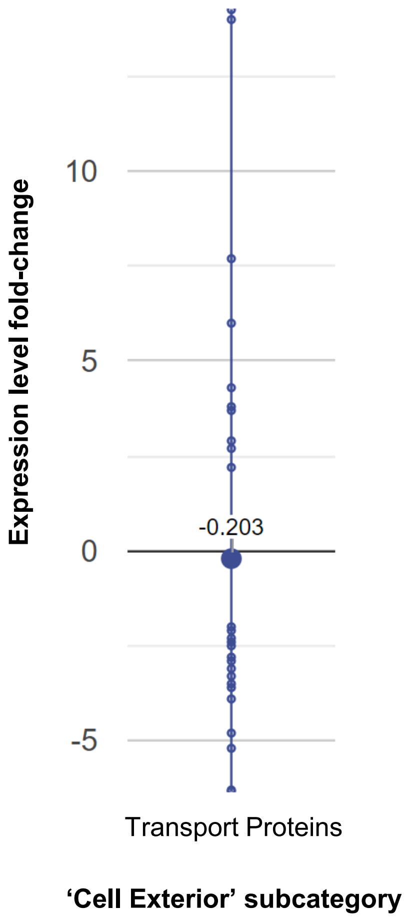

B)

'Cell Exterior' subcategory	Locus tag	Protein (gene)	Expression level fold-change
Transport Proteins	N5F16_22000	4-amino-4-deoxy-L-arabinose-phosphoundecaprenol flippase subunit ArnE (arnE)	14.26
Transport Proteins	N5F16_08425	magnesium transporter (N5F16_08425)	7.71
Transport Proteins	N5F16_21995	4-amino-4-deoxy-L-arabinose-phosphoundecaprenol flippase subunit ArnF (arnF)	6.04
Transport Proteins	N5F16_02860	magnesium-translocating P-type ATPase (mgtA)	4.27
Transport Proteins	N5F16_01500	maltose/maltodextrin ABC transporter ATP-binding protein MalK (malK)	3.76
Transport Proteins	N5F16_16175	PTS cellobiose transporter subunit IIC (celB)	3.68
Transport Proteins	N5F16_02850	PTS trehalose transporter subunit IIBC (treB)	2.88
Transport Proteins	N5F16_09990	outer membrane lipoprotein SlyB (slyB)	2.73
Transport Proteins	N5F16_01495	maltose/maltodextrin ABC transporter substrate-binding protein MalE (malE)	2.69
Transport Proteins	N5F16_01490	maltose ABC transporter permease MalF (malF)	2.16
Transport Proteins	N5F16_09320	thiamine ABC transporter permease	-2
Transport Proteins	N5F16_18785	PTS mannitol transporter subunit IICB	-2.09
Transport Proteins	N5F16_07480	putrescine ABC transporter permease PotH (potH)	-2.32
Transport Proteins	N5F16_17720	PTS glucitol/sorbitol transporter subunit IIC (srlA)	-2.35
Transport Proteins	N5F16_17730	PTS glucitol/sorbitol transporter subunit IIA (srlB)	-2.37
Transport Proteins	N5F16_07470	spermidine/putrescine ABC transporter substrate-binding protein PotF (potF)	-2.52
Transport Proteins	N5F16_07485	putrescine ABC transporter permease PotI (potI)	-2.54
Transport Proteins	N5F16_07475	putrescine ABC transporter ATP-binding subunit PotG (potG)	-2.84
Transport Proteins	N5F16_17725	PTS glucitol/sorbitol transporter subunit IIB (N5F16_17725)	-2.93
Transport Proteins	N5F16_20040	autoinducer 2 ABC transporter ATP-binding protein LsrA (lsrA)	-3.12
Transport Proteins	N5F16_06635	potassium-transporting ATPase subunit KdpA (kdpA)	-3.33
Transport Proteins	N5F16_07515	arginine ABC transporter permease ArtM (artM)	-3.51
Transport Proteins	N5F16_20030	autoinducer 2 import system permease LsrD	-3.6
Transport Proteins	N5F16_06630	potassium-transporting ATPase subunit KdpB (kdpB)	-3.9
Transport Proteins	N5F16_20035	autoinducer 2 ABC transporter permease LsrC (lsrC)	-4.77
Transport Proteins	N5F16_17770	HoxN/HupN/NixA family nickel/cobalt transporter	-5.15
Transport Proteins	N5F16_06600	putrescine-ornithine antiporter (potE)	-6.33

FIG S13 Differentially expressed genes (DEGs) representing the 'Cell Exterior' category in Eh4236Csa13R isolate; (panel A) graph depicting the average (the large blue dots) and individual (the large blue dots) gene expression level fold-change values for DEGs belonging to specific 'Cell Exterior' subcategories, (panel B) table with characteristics of the DEGs used to create the graph. Analysis was performed using the 'Omics' module of Pathway Tools v26.0 software (16).

Differentially expressed genes (DEGs) representing the 'Regulation' category in Eh4236Csa13R isolate

A)

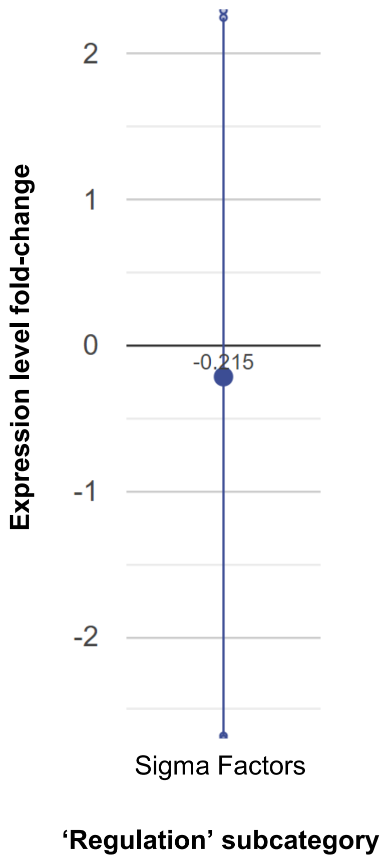

B)

'Regulation' subcategory	Locus tag	Protein (gene)	Expression level fold-change
Sigma Factors	N5F16_14065	RNA polymerase sigma factor FliA	2.25
Sigma Factors	N5F16_16620	RNA polymerase sigma factor RpoE (rpoE)	-2.68

FIG S14 Differentially expressed genes (DEGs) representing the 'Regulation' category in Eh4236Csa13R isolate; (panel A) graph depicting the average (the large blue dots) and individual (the large blue dots) gene expression level fold-change values for DEGs belonging to specific 'Regulation' subcategories, (panel B) table with characteristics of the DEGs used to create the graph. Analysis was performed using the 'Omics' module of Pathway Tools v26.0 software (16).

Differentially expressed genes (DEGs) representing the ‘Objects Not Present in any Subsystem’ category in Eh4236Csa13R isolate

A)

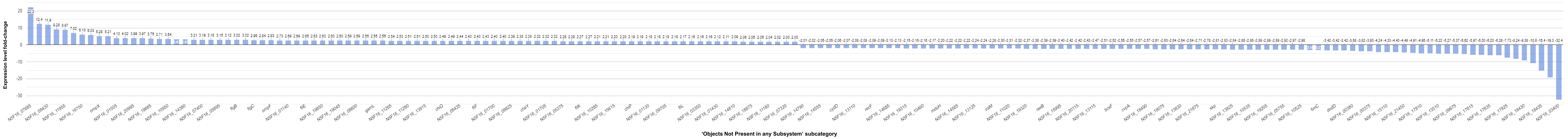

FIG S15 Differentially expressed genes (DEGs) representing the ‘Objects Not Present in any Subsystem’ category in Eh4236Csa13R isolate; (panel A) graph depicting the average (the large blue dots) and individual (the large blue dots) gene expression level fold-change values for DEGs belonging to specific ‘Objects Not Present in any Subsystem’ subcategories, (panel B, next page) table with characteristics of the DEGs used to create the graph. Analysis was performed using the ‘Omics’ module of Pathway Tools v26.0 software (16).

Differentially expressed genes (DEGs) representing the ‘Objects Not Present in any Subsystem’ category in Eh4236Csa13R isolate, cont.

B)

Locus tag	Protein (gene)	Expression level fold-change
N5F16_07685	Virk/Ybjx family protein	22.3
N5F16_22010	4-deoxy-4-formamido-l-arabinose- phosphoundecaprenol deformylase	12.41
N5F16_08430	Deda family protein	11.91
N5F16_13585	Virk/ybjx family protein	9.28
N5F16_11955	MFS transporter	8.97
N5F16_03370	Yfaz family protein	7.02
N5F16_16750	SMP-30/gluconolactonase/LRE family protein	6.1
N5F16_07250	Efflux RND transporter periplasmic adaptor subunit	6.03
N5F16_07180	Outer membrane protein ompx	5.28
N5F16_11270	Ig-like domain-containing protein	5.21
N5F16_01505	Maltoporin	4.13
N5F16_10945	Extracellular solute-binding protein	4.02
N5F16_20995	Alpa family phage regulatory protein	3.99
N5F16_20970	Hypothetical protein	3.97
N5F16_19885	GPO family capsid scaffolding protein	3.75
N5F16_08605	Pirin family protein	3.71
N5F16_10950	ABC transporter permease subunit	3.54
N5F16_02220	Arac family transcriptional regulator	3.47
N5F16_14380	Acyltransferase family protein	3.43
N5F16_00060	MFS transporter	3.21
N5F16_07400	Phosphatase PAP2 family protein	3.19
N5F16_10235	Toxin ydat domain-containing protein	3.18
N5F16_05895	Cation-transporting p-type atpase	3.15
N5F16_20990	Hypothetical protein	3.12
N5F16_08805	Flagellar basal body rod protein flgb	3.02
N5F16_19880	Phage major capsid protein, P2 family	3.02
N5F16_08810	Flagellar basal body rod protein flgc	2.96
N5F16_14680	Polysaccharide export protein	2.84
N5F16_07920	Porin ompf	2.83
N5F16_08815	Flagellar hook assembly protein flgd	2.73
N5F16_01140	Sugar phosphate isomerase/epimerase	2.69
N5F16_19875	Phage terminase small subunit	2.69
N5F16_14100	Flagellar hook-basal body complex protein flie	2.65
N5F16_15610	TIGR01777 family oxidoreductase	2.63
N5F16_19850	DUF2597 family protein	2.63
N5F16_07085	Transcriptional regulator cecr	2.6
N5F16_19045	2-hydroxycarboxylate transporter family protein	2.6
N5F16_03225	Amidohydrolase	2.59
N5F16_08600	Isochorismatase family protein	2.59
N5F16_07260	Efflux transporter outer membrane subunit	2.55
N5F16_10180	Host nuclease inhibitor gaml	2.55
N5F16_22400	Helix-turn-helix domain-containing protein	2.55
N5F16_11265	Tolc family outer membrane protein	2.54
N5F16_13330	Flagellar brake protein	2.53
N5F16_11280	Hlyd family type I secretion periplasmic adaptor subunit	2.51
N5F16_19840	M15 family metallopeptidase	2.51
N5F16_13915	Anaerobic c4-dicarboxylate transporter	2.5
N5F16_18870	Hypothetical protein	2.5
N5F16_06565	Chiq/ybfn family lipoprotein	2.49
N5F16_20945	Phage portal protein	2.49
N5F16_08435	SDR family oxidoreductase	2.44
N5F16_08800	Flagellar basal body p-ring formation chaperone flga	2.43
N5F16_14105	Flagellar basal-body ms-ring/collar protein flif	2.43
N5F16_19890	Terminase family protein	2.43
N5F16_01700	Glycoside hydrolase family 19 protein	2.4
N5F16_02135	Phosphonate metabolism transcriptional regulator phnf	2.4
N5F16_08825	Flagellar basal body rod protein flgf	2.39
N5F16_10185	Cell division protein ftsz	2.38
N5F16_13790	Chemotaxis response regulator chey	2.38
N5F16_00510	Virulence rhum family protein	2.32
N5F16_01705	Hypothetical protein	2.32
N5F16_12795	Dna-binding transcriptional regulator	2.32
N5F16_05375	Sgrr family transcriptional regulator	2.28
N5F16_10245	DNA replication protein	2.28
N5F16_14130	Flagellar hook length control protein flik	2.27
N5F16_19040	Fumarylacetoacetate hydrolase family protein	2.27
N5F16_10285	DUF1364 domain-containing protein	2.21
N5F16_19820	Phage tail tape measure protein	2.21
N5F16_15615	GNAT family n-acetyltransferase	2.2
N5F16_20950	HK97 family phage prohead protease	2.2
N5F16_06560	Chitoporin	2.19
N5F16_18320	PTS transporter subunit EIIC	2.19
N5F16_01130	MFS transporter	2.18
N5F16_01135	Sugar kinase	2.18
N5F16_09705	Merr family transcriptional regulator	2.18
N5F16_19845	Phage holin family protein	2.18
N5F16_14135	Flagellar basal body-associated protein flil	2.17
N5F16_01635	Hypothetical protein	2.16
N5F16_03350	Brna antitoxin family protein	2.16
N5F16_08840	Flagellar basal body p-ring protein flgi	2.16
N5F16_01430	Type II toxin-antitoxin system rele/pare family toxin	2.12
N5F16_11445	Transcriptional regulator ftra	2.11
N5F16_14810	Mannitol dehydrogenase family protein	2.09
N5F16_12075	Cupin domain-containing protein	2.06
N5F16_18875	Non-heme iron oxygenase ferredoxin subunit	2.05
N5F16_20955	Phage major capsid protein	2.05
N5F16_11180	DMT family transporter	2.04
N5F16_16185	Leucyl aminopeptidase family protein	2.02
N5F16_07320	Glycyl-radical enzyme activating protein	2.0
N5F16_19855	DUF2586 domain-containing protein	2.0
N5F16_14790	Mandelate racemase family protein	-2.01
N5F16_13190	Type VI secretion system atpase tssh	-2.02
N5F16_14555	Oligosaccharide repeat unit polymerase	-2.05
N5F16_16440	Co-chaperone hscb	-2.05
N5F16_07730	Cysteine/glutathione ABC transporter permease/atp-binding protein cydd	-2.06
N5F16_00960	Tyrosine recombinase xerc	-2.07
N5F16_13110	Hypothetical protein	-2.08
N5F16_14540	Oligosaccharide flippase family protein	-2.08
N5F16_00015	DNA replication/repair protein recf	-2.09
N5F16_13130	Competence protein comj	-2.09
N5F16_14685	Terc family protein	-2.1
N5F16_03380	Hypothetical protein	-2.13
N5F16_19315	N5f16_19315	-2.15
N5F16_00880	ECA oligosaccharide polymerase	-2.16
N5F16_10460	Glucosyltransferase domain-containing protein	-2.16
N5F16_16795	50S ribosomal protein L19	-2.17
N5F16_08695	Glucans biosynthesis glucosyltransferase mdoh	-2.2
N5F16_13235	Type VI secretion system amidase immunity protein tai4	-2.22
N5F16_14565	Glycosyltransferase	-2.22
N5F16_18155	DUF423 domain-containing protein	-2.22
N5F16_13125	Hypothetical protein	-2.24
N5F16_13145	Competence protein comj	-2.24
N5F16_10540	Anti-adapter protein iram	-2.28
N5F16_21145	50S ribosomal protein L17	-2.3
N5F16_11020	GGDEF domain-containing protein	-2.31
N5F16_20820	Rod shape-determining protein mred	-2.32
N5F16_19320	EAL domain-containing protein	-2.37
N5F16_13290	Type VI secretion system baseplate subunit tssk	-2.38
N5F16_16610	Sigma-e factor regulatory protein rseb	-2.39
N5F16_19395	N5f16_19395	-2.39
N5F16_15995	Rpoe-regulated lipoprotein	-2.4
N5F16_18780	Zinc-binding dehydrogenase	-2.42
N5F16_20115	DUF1090 domain-containing protein	-2.42
N5F16_04550	Sigma E protease regulator rsep	-2.43
N5F16_13115	Hypothetical protein	-2.47
N5F16_22625	Yijd family membrane protein	-2.51
N5F16_22210	Cellulose biosynthesis protein bcsf	-2.52
N5F16_11585	Hypothetical protein	-2.55
N5F16_15655	Colicin V production protein	-2.55
N5F16_13105	N5f16_13105	-2.57
N5F16_18490	SDR family oxidoreductase	-2.57
N5F16_09670	Fimbrial protein	-2.61
N5F16_19075	Hybrid sensor histidine kinase/response regulator	-2.63
N5F16_05745	Type 1 fimbrial protein subunit fimi	-2.64
N5F16_13830	Spore coat U domain-containing protein	-2.64
N5F16_16605	Soxr-reducing system protein rsec	-2.64
N5F16_21875	TRAP transporter large permease	-2.71
N5F16_18445	Manganese catalase family protein	-2.78
N5F16_04560	Molecular chaperone skp	-2.81
N5F16_09500	50S ribosomal protein L35	-2.83
N5F16_13825	Molecular chaperone	-2.84
N5F16_07905	Ycbk family protein	-2.86
N5F16_10535	Cold shock domain-containing protein	-2.86
N5F16_09505	50S ribosomal protein L20	-2.89
N5F16_19205	Hypothetical protein	-2.89
N5F16_20870	Yhdt family protein	-2.89
N5F16_05755	Fimbrial biogenesis usher protein	-2.92
N5F16_06335	Transcription antiterminator/RNA stability regulator cspe	-2.97
N5F16_10525	Hypothetical protein	-2.98
N5F16_03135	DNA sulfur modification protein dndd	-3.18
N5F16_05750	Type 1 fimbria chaperone fimc	-3.27
N5F16_00370	Hypothetical protein	-3.42
N5F16_03140	DNA sulfur modification protein dndd	-3.42
N5F16_13635	Formate-dependent phosphoribosylglycinamide formyltransferase	-3.42
N5F16_00380	Hypothetical protein	-3.68
N5F16_07910	MBL fold metallo-hydrolase	-3.82
N5F16_00375	Hypothetical protein	-3.93
N5F16_03385	O-antigen ligase family protein	-4.24
N5F16_15110	Yeih family protein	-4.33
N5F16_17865	Hydrogenase expression/formation protein hype	-4.4
N5F16_21450	Aspartate aminotransferase family protein	-4.49
N5F16_07510	ABC transporter substrate-binding protein artj	-4.91
N5F16_17810	Nadh-quinone oxidoreductase subunit B family protein	-4.95
N5F16_17805	Formate hydrogenlyase maturation hych family protein	-5.11
N5F16_13510	N5f16_13510	-5.22
N5F16_17845	Hydrogenase maturation nickel metallochaperone hypa	-5.27
N5F16_09675	Luxr c-terminal-related transcriptional regulator	-5.37
N5F16_17450	Ig-like domain-containing protein	-5.62
N5F16_17815	Formate hydrogenlyase complex iron-sulfur subunit	-5.97
N5F16_17850	Hydrogenase nickel incorporation protein hypb	-6
N5F16_17835	4fe-4s dicluster domain-containing protein	-6.2
N5F16_17775	Carbamoyltransferase hypf	-6.28
N5F16_17825	Respiratory chain complex I subunit 1 family protein	-7.73
N5F16_17855	Hypc/hybg/hupf family hydrogenase formation chaperone	-8.24
N5F16_18430	General stress protein	-9.38
N5F16_03395	Ybdd/yjix family protein	-10.8
N5F16_18435	Ferritin-like domain-containing protein	-15.42
N5F16_18440	Ferritin-like domain-containing protein	-19.32
N5F16_03400	Pyruvate transporter YjiY	-32.41

FIG S15 cont. Differentially expressed genes (DEGs) representing the ‘Objects Not Present in any Subsystem’ category in Eh4236ColR isolate; (panel A, previous page) graph depicting the average (the large blue dots) and individual (the large blue dots) gene expression level fold-change values for DEGs belonging to specific ‘Objects Not Present in any Subsystem’ subcategories, (panel B) table with characteristics of the DEGs used to create the graph. Analysis was performed using the ‘Omics’ module of Pathway Tools v26.0 software (16).
